# Editorial for Special Topics: Imaging-Based Diagnosis for Prostate Cancer—State of the Art

**DOI:** 10.3390/diagnostics14182016

**Published:** 2024-09-12

**Authors:** Rulon Mayer, Peter L. Choyke, Charles B. Simone II

**Affiliations:** 1 Department of Radiation Oncology, Perelman School of Medicine, University of Pennsylvania, Philadelphia, PA 19104, USA; 2 Oncoscore, Garrett Park, MD 20896, USA; 3 National Institutes of Health, Bethesda, MD 20892, USA; pchoyke@mail.nih.gov; 4 New York Proton Center, New York, NY 10035, USA; csimone@nyproton.com

## 1. Introduction

This Special Topics Issue, “Imaging-based Diagnosis of Prostate Cancer—State of the Art”, of *Diagnostics* compiles 10 select articles [1,2,3,4,5,6,7,8,9,10] describing current advances in detecting and assessing prostate tumors using imaging. Seven articles [1,2,3,6,7,9,10] summarize studies of multi- or bi-parametric MRI for assessing prostate tumors and determining if they are likely to represent clinically significant prostate cancer (CsPCa). The studies that comprise the Special Topics series employ both subjective visual assessments by trained radiologists as well as more objective determinants employing quantitative procedures and algorithms. In addition, three articles [4,5,8] summarize recent advances in targeting prostate-specific membrane antigen (PSMA) using PET/CT to improve metastasis detection.

## 2. Background

“A generation which ignores history has no past—and no future”, Robert Heinlein

Accurate, timely evaluation of a patient suspected of harboring cancer leads to early optimal management of the disease with better outcomes [11,12]. A part of the assessment [11,12] typically involves determining the presence or absence of the disease, the aggressiveness of the disease, and to what extent the disease has metastasized beyond the primary site. Early assessment of a cancer can lead to timely therapy and thereby increased likelihood of effective disease control [13]. If the tumor is still localized, treatments such as surgery and radiation therapy are possibly curable, whereas if the disease has metastasized, systemic therapy may be required and prognosis worsens. Optimally, the diagnostic pathway should be consistent with existing medical workflows, economical, efficient, and reliable while posing little risk to the patient.

Conventional evaluations for prostate cancer have relied on prostate-specific antigen (PSA) measurements followed by systematic or random prostate biopsies [14]. Although PSA screening tests are convenient and readily available, PSA suffers from poor specificity and accuracy [15]. Specifically, many benign conditions elevate PSA, while some malignant conditions fail to elevate PSA. Adding clinical factors, such as patient age, patient ethnicity, and prostate size, slightly improve the diagnostic performance relative to PSA measurement alone, but the strategy of PSA screening continues to suffer from non-specificity and insensitivity [16]. Prior to the use of MRI, ultrasound was used to guide needle biopsies [17], followed by pathology examination of the extracted tissues. However, ultrasound was inadequate in properly localizing lesions and, as a result, normal regions were oversampled, leading to over-diagnosis, and abnormal regions were undersampled, leading to under-diagnosis.

The advent of routine prostate MRI to localize prostatic tumors has changed the diagnostic pathway of prostate cancer [18]. MRI of the prostate can identify lesions into which needle biopsies can be directed, thus improving sampling accuracy. However, the interpretation of prostate MRIs remains subjective, and historically, there has been no standard lexicon for describing lesions on prostate MRI. Relatively recently, the Prostate Imaging Reporting and Data System (PI-RADS) protocol was introduced to standardize prostate MRI reporting and assign the risk of csPCa for each identified lesion [19]. However, consistent class assignments depend on the experience and training of the radiologists [20], and inter-reader disagreements are common.

To reduce inconsistent evaluations resulting from visual inspection of MRI, a more quantitative approach to evaluating prostate tumor has been investigated. Specifically, machine learning and neural networking employing radiomics and spatial features [21] have been applied to prostate MRI to determine the likelihood of a csPCa. In contrast, recently [6], spectral/statistical algorithms adapted from remote sensing have been applied to spatially registered MRI to assess prostate cancer.

Until recently, the determination of prostate cancer metastases has depended on conventional bone scans and computed tomography (CT) [22]. Recently, a new positron emitting radionuclide, conjugated to a prostate-specific membrane antigen (PSMA)-targeting ligand, has resulted in a highly sensitive tool for assessing metastatic disease. PSMA-PET/CT is now commonly employed to stage prostate cancers or identify recurrences [23]. Such advances significantly improve the detection of nodal or bony metastases.

## 3. Results

Table 1 lists the papers comprising the Special Topics. The column headings in Table 1 lists features of the papers, such as the imaging modality, the use of PI-RADS goals, the type of algorithm, metastases, morphology, the number of patients or lesions, and any significant results. The MRI articles discuss using MRI to evaluate prostate tumors. The PET/CT scan papers examine topics detecting prostate cancer metastases.

Seven papers used MRI to evaluate prostate tumors. Barone [1], Bertelli [2], Tomioka [9], and Volz [10] employed MRI and PI-RADS to help assess the likelihood of csPCa. Mayer [7], Tomioka [9], and Volz [10] studied morphology, in particular tumor geometry, and tumor or prostate volume and their role in determining tumor aggressiveness. Significantly, MRI volume measurements tend to underestimate actual tumor volume based on histology volumetrics, as is discussed by Mayer [7] and Tomioka [9].

Quantitative assessments using algorithms analyzed MR images to predict the prostate tumor grade. Dominguez [3] employed machine learning and radiomics to determine prostate tumor’s aggressiveness. Mayer [6,7] used a tumor’s spectral signal-to-clutter ratio and the tumors’s eccentricity and volume, respectively, to extend previous spectral/statistical approaches applied to spatially registered multi-parametric MRI using contrast material to the bi-parametric MRI with no contrast material.

Three papers (Gandini [4], Lee [5], Rovera [8]) employed PSMA PET/CT for staging prostate cancer. These papers demonstrated improved prostate cancer metastasis detection. Gandini [4] applied a recent PSMA PET/CT to find lesions in the heart from prostate cancer metastases, confirming an earlier finding for a new site. Lee [5] compared chelating agents (DOTA vs. NOTA) for ^68^Ga-labeled PSMA PET/CT targeting and observed that NOTA achieved greater tumor and lower liver uptake in human and mouse sera and xenografts. Rovera [8] tested the feasibility of using machine learning to automatically segment nodes disclosed on PSMA PET/CT for future intraoperative procedures. Rovera [8] showed promising results for timely semi-quantitative analysis of PET/CT images in the operating room to aid treatment.

## 4. Discussion: Future Trends

“It is difficult to make predictions, especially about the future.” Yogi Berra

This Special Topics issue provides a “snapshot” into current research areas in prostate cancer diagnostic imaging. This compilation offers an opportunity to reflect and speculate about future directions.

This Special Topics follows the recent trend of evaluating prostate tumors using quantitative evaluation and application of mathematical algorithms in MRI. If implemented, patients should receive more consistent and accurate assessments with reduced intra-reader variability. Moreover, such algorithms can be used to assess the quality of the MRI and whether it is suitable for interpretation.

The following suggests **shorter-term, future** advances that directly connect to this Special Topics issue and to the current literature.

**Resolving Prostate Tumor Volume MRI Disparity Measurement Issues**: Regarding tumor volume measurements [24], Tomioka [9] and Mayer [7] researched the disparity between MRI and histology analysis of prostatectomy. This topic is important for more accurately treating a prostate cancer patient with focal therapy, including surgery and radiation therapy, and for proper determination of tumor margin for possible tumor-directed boosts in radiation treatment.

**Large Patient Protocol Studies of Multi-Parametric MRI vs. Bi-Parametric MRI**: Bi-parametric (BP) MRI scanning simplifies the evaluation, reduces the scanning time, increases the clinic patient throughput, and reduces possible patient side effects relative to injecting contrast material for multi-parametric MRI (Mayer [6]. However, MP-MRI reveals the tumor vasculature and more information regarding the tumor architecture, and it may be needed to accurately assess a prostate tumor. BP-MR is more amenable to algorithm developments because contrast-enhanced MRI involves repeated imaging at different times and is difficult to standardize.

**Qualitative/Quantitative Color Maps:** Color, instead of the standard monochrome greyscale, may be used to visually inspect individual images to discern and interpret lesions. The coloring scheme assigns red, green, and blue to channels in spatially registered MRI to form a composite color image. In this case, different colors are used to display PCa and normal tissue. Furthermore, these color images can also be quantified [24,25,26]. This coloring does **not** equate to false or pseudo coloring applied to individual images. Such false color displays show relative intensities within a given image. Future research [26] is indicated to clinically test the value of tumor color display for patient care management and possibly derive new quantitative metrics for assessing tumors. In addition, the coloring scheme may reveal tumor heterogeneity, such as the presence of necrosis, inflammation, and tumor habitats and microstructure.

**Cross-Clinic Transformation**: MRI scanning conditions (magnetic field strength, pulse sequences, etc.) that affect the quality of the image can vary among clinics. Such variations hinder AI-based approaches for clinical implementation because of the diversity of appearances. Previously [27], “whitening–dewhitening”-transformed target signatures based on Gleason score status for supervised target detections were employed to handle the changes in conditions. Future research may transform prostate tumor signatures across multiple clinics. A single library may hold multiple tumor signatures in the future.

**Artificial Intelligence vs. Spectral/Statistical Algorithms:** This Special Topics issue compiled examples of algorithms and quantitative approaches applied to MRI, such as artificial intelligence and spectral/statistical techniques. Dominguez [3] used spatial features such as textures in their machine learning application. Mayer [6,7] applied spectral/statistical algorithms to spatially registered MRI in order to assess prostate tumors. A direct quantitative comparison between the two techniques involving a large patient cohort is merited. Adding spatial textures to the increasing number of dimensions of bi-parametric hypercubes also merits investigation.

The following suggests **longer-term, speculative future** advances.

**New Biomarkers**: New biomarkers, beyond PSA [28,29], show promise in identifying the presence of prostate tumors with fewer false positives than PSA. Future studies might combine these novel biomarkers with PI-RADS, MP-MRI, and/or Bi-MRI for further improvements.

**Focal Proton Therapy:** Proton beam therapy more precisely delivers radiation therapy [30] to its target, sparing normal tissue. Improved imaging [31] may reveal that certain patients might benefit from exposing only a portion of the prostate, rather than the current standard practice of the entire prostate, to irradiation, thus reducing possible side effects from unnecessarily exposing nearby normal tissues to irradiation. At present, the feasibility of using MP-MRI for focal radiation therapy [30,31] has only been shown through treatment planning studies [32]. Focal treatment directed at metastases may also be combined with enhanced CT/PET/PSMA scanning.

**MP-MRI and Genomics**: The combination of imaging and tumor genomics is a particularly potent tool for predicting outcomes. A meta-analysis [33] found that MP-MRI-visible cancers are associated with proliferative signaling, DNA damage, and inflammatory processes. Others [34,35] correlated MP-MRI features with aggressive genomic and proteomic features. Further research incorporating all MP-MRI modalities may add additional value to genomic metrics.

**Magnetic Resonance Spectroscopy (MRS):** MRS, like airborne hyperspectral imagers, uses many bands. However, MRS suffers from poor spatial resolution (the resolution of MRS [36] is 0.25 cm^3^, while that of MP-MRI is 0.006 cm^3^), resulting in sampling issues. The limited sampling reduces the ability to discriminate tumors from normal tissue. The limited MRS sampling precludes exploiting the statistical analysis due to the background covariance matrix inversion non-singularity. Covariance matrix regularization can mitigate the insufficient sampling. Hardware and software developments may sufficiently elevate the MRS spatial resolution by degrading the spectral resolution, which may enable MRS statistical analysis, as in remote sensing. Concepts developed for remote sensing proved the value of making such trade-offs and could be applied to the clinical diagnosis of prostate cancer.

## Figures and Tables

**Table 1:** Summary of articles in “Imaging-based Diagnosis of Prostate Cancer—State of the Art”.

Author	Imager	PI-RADS	Goal	Algorithm	MP-MRI/ BP-MRI	Metastases	Prostate/Tumor Size/Shape	#, Samples	Significant Results
Barone [1]	MRI	X	CsPCa		X			389	MP-MRI similar to Biopsy
Bertelli [2]	MRI	X	CsPCa		X		X	104 lesions	ADC threshold, Sensitivity = 0.86, Specificity = 0.59
Dominguez [3]	MRI		CsPCa	ML/Radiomics			X	86	AUC: 0.80
Gandini [4]	PET/CT/PSMA		Detection			X		1	Novel Metastasis Site: Heart
Lee [5]	PET/CT/PSMA		Detection			X		Cells, Animal	Chelate: NOTA > DOTA
Mayer [6]	MRI		CsPCa	Spectral/Statistics	X			42	AUC: 1.0 [1.0–1.0]
Mayer [7]	MRI		CsPCa	Spectral/Statistics	X		X	42	AUC: 0.45–0.96
Rovera [8]	PET/CT/PSMA		Detection	Segment/ML		X		6	Segmentation Precision (=97–99%), Recall (=68–81%)
Tomioka [9]	MRI	X	CsPCa	pTB	X		X	162	Tumors grow beyond MRI
Volz [10]	MRI	X	PI-RADS/Detect/CsPCa		X		X	1039	Prostate Volume affects Detection

Abbreviations: MP-MRI, multi-parametric MRI; BI-MRI, bi-parametric MRI; PET, positron emission tomography; CT, computed tomography; PI-RADS, Prostate Imaging Reporting and Data System; PSA, prostate-specific antigen; CsPCa, clinically significant prostate cancer; AUC, area under the curve; ML, machine learning; pTB, perilesional targeted biopsy; #, number.
